# Changes in the basic birth beliefs following the first birth experience: Self-fulfilling prophecies?

**DOI:** 10.1371/journal.pone.0208090

**Published:** 2018-11-26

**Authors:** Heidi Preis, Joseph Pardo, Yoav Peled, Yael Benyamini

**Affiliations:** 1 Bob Shapell School of Social Work, Tel Aviv University, Tel Aviv, Israel; 2 Department of Obstetrics and Gynecology, Helen Schneider Hospital for Women, Rabin Medical Center–Beilinson Hospital; affiliated with Sackler Faculty of Medicine, Tel Aviv University, Tel Aviv, Israel; Monash University, AUSTRALIA

## Abstract

Women's basic beliefs about birth as a natural and as a medical process are associated with childbirth choices and experience. These beliefs have only recently been quantified and not much is known about their development. In the current study, we assessed the differential effects of the objective and the subjective birth experience on changes in these beliefs. Using self-report questionnaires, we evaluated prenatal to postpartum changes among 342 Israeli first-time mothers. Participants were recruited during pregnancy, between February 2016 and January 2017, mostly in clinical settings, and followed-up two months postpartum. On average, women's beliefs about birth being natural weakened following childbirth and their belief about birth being medical strengthened. In regression models, it was either the objective or the subjective experience that was related to change in the basic birth beliefs: A more medicalized birth was associated with strengthening of the medical belief while greater birth satisfaction was related to strengthening of the natural belief. A mediation effect was observed, which indicated that the beliefs are strengthened when the lived experience fulfilled women's expectation about birth being satisfying, natural or medical. This study adds to the growing body of knowledge regarding the development and evolution of the birth beliefs. It highlights the need to view the beliefs separately and to distinctively assess the objective and subjective birth experience. It supports the need to empower mothers, especially those who had more medicalized births or unsatisfactory ones, which would help conserve their belief in their body and in the normal physiological course of birth.

## Introduction

People's decision-making regarding health-related issues is heavily based on their beliefs and perceptions [1]. The beliefs and perceptions affect not only health behaviors but also health outcomes [2]. The aftermath of a health event or threat, in turn, is related not only to its objective severity, but also to the way it is subjectively experienced [3]. The experience will go on to affect people's perceptions [4]. This reciprocal pattern of perception-behavior-outcome-cognitive appraisal-perception can be used as a framework to understand women's basic birth beliefs. In the current study we examined how birth beliefs expressed during pregnancy relate to women's objective and subjective birth experience and how the birth experiences differentially affect postpartum birth beliefs.

The basic birth beliefs refer to the way women view the physical aspects of birth. They are composed of a medical birth belief and a natural birth belief and women may have varying views regarding each belief. The medical and natural beliefs are (negatively) correlated but only moderately, so they do not form a dichotomous continuum. The two beliefs are independent concepts and are not mutually exclusive [5,6]. Beliefs that birth is a medical process relate to viewing it as risky, that it should be performed under medical supervision and that the pain of birth is needless. Beliefs that birth is a natural process imply that it is safe, women's bodies know how to give birth, and birth should not be interfered with [6].

Qualitative studies have shown that perceiving birth to be natural and safe or medical and risky were related to more medicalized birth choices, such as cesarean delivery (CD) or using analgesia during labor, or more natural birth choices, such as home birth, giving birth at free-standing birth centers or delivering without epidural analgesia [7–10]. The pivotal role of the birth beliefs in determining women's birth preferences [11] and their consequent birth (both planned and emergency) [12] was also recently corroborated in two longitudinal studies. The beliefs are closely linked to other perceptions of birth, such as fear of childbirth and the subjective birth experience [11,13,14]. These studies suggest that the beliefs are central contributors to the physical and emotional birth experience, therefore it is important to study how they evolve. This is especially important among first-time mothers, whose birth perceptions are more susceptible to change [15].

The beliefs have only recently been quantified and there is a dearth of empirical knowledge regarding their development. Nonetheless, there is evidence that women who had a more medicalized obstetric history (such as pregnancy loss) have a stronger belief that birth is a medical process and a weaker belief that it is a natural process [6]. In addition, evidence suggests that the birth experience is related to postpartum cognitive and affective responses. The birth experience is made up of two distinct yet intertwined constructs: the objective or actual birth (i.e. mode of delivery, interventions during birth) as well as the subjective birth experience (i.e. the cognitive appraisal or emotional experience of birth) [16]. In their recent study, Haines et al. [14] found that having a previous negative birth experience and having a CD are related to viewing birth as less natural, suggesting that perhaps the lived birth experience might affect women's perception of birth. Several prospective studies have found that place and mode of birth (i.e. CD, home birth, vaginal delivery [VD] with or without epidural, birth center) and satisfaction with birth to be related to changes in women's perceptions and emotions after birth. For example, having an emergency CD was found to be associated with an increase in fear of birth [17,18] and lower birth satisfaction was found to be related to an increase in postpartum depression [19,20]. Those studies suggest that perceptions regarding birth can change following the objective or the subjective birth experience.

In the current study we wished to assess the effects of actual and subjective birth experiences on first-time mothers' basic birth beliefs. To our knowledge, an investigation studying changes in the beliefs following birth has not been documented. Gaining deeper insight into the mechanisms related to the beliefs will propel theoretical knowledge regarding birth and could also have clinical implications. Prenatally, understanding women's beliefs and their correlates can help instigate open discussion with women about their expectations regarding childbirth and promote patient-centered care and shared-decision making regarding birth choices. Postpartum, understanding how the lived birth experience affects women's beliefs could help practitioners relate to women's experiences and ameliorate perceptual consequences that a negative birth experience might have. Since perceptions about birth are known to develop even before the first pregnancy [21], we expect postpartum beliefs to be predicted mostly by their prenatal levels. However, we assume that changes in the beliefs could take place following childbirth and expect that more medicalized birth and or a more negative subjective experience will be related to strengthening of the medical birth beliefs and weakening of the natural birth belief.

## Methods

### Participants

The current report focuses on 342 primiparae who completed questionnaires prenatally (T1 *M =* 31.7±5.2 weeks gestation) and two months postpartum (T2 *M =* 9.2±3.6 weeks postpartum). Eligibility criteria included a singleton pregnancy ≥ 24 weeks gestation, with VD medically possible (for example no placenta previa). Exclusion criteria were being in labor pain (VAS > 3) or having a medical emergency and non-Hebrew speaking.

### Procedure

The study was approved by the Research Ethics Committees of all participating institutions (Clalit Health Services- 120-15-COM2; Rabin Medical Center- 339-15-RCM; Tel Aviv University Institutional Review Board). Recruitment of women for the study took place between February 2016 and January 2017 at three settings: (1) At four different Women’s Health Centers of Clalit Health Care Services in the center of Israel while waiting for prenatal check-up; (2) At Rabin Medical Center, (Large hospital) when coming for a prenatal class, hospital tour, check-up or birth; (3) Purposeful sampling of women who preferred to birth in alternative modes of birth (e.g., home births or natural birth centers) through specific, natural/home birth Facebook groups, home midwives, or personal acquaintances.

Recruitment was mostly done by trained social work graduate students (at the Women's Health Centers and in the alternative sampling) with a small part conducted by midwives (at the hospital). Women who were recruited at the clinical settings received an explanation about the study from a member of the study team and were asked for their written consent. Participants were also asked for their contact information for follow-up. Thereafter, they completed the baseline questionnaire (T1). Women recruited in the purposeful, alternative fashion, were offered a paper questionnaire or an identical online version. Before completing the questionnaire, these women were also asked to indicate their consent and provide their contact information.

Overall, 976 primiparae and multiparae women completed the baseline questionnaire: Sixty-six percent were recruited at Women's Health Centers, 22% in the alternative sampling and 12% at the hospital. Out of 1,059 women who were approached at the clinical settings and were eligible to participate in the study, 764 agreed to participate and filled out the first questionnaire (72.6% recruitment rate). Main reasons for not participating were disinterest, dislike of surveys, concerns about anonymity and lack of time. Two of the recruited women were later excluded because of perinatal infant mortality. Another 214 women were recruited in the alternative sampling. Since the invitation to the survey in the alternative sample was mostly online, we were unable to determine recruitment rates. Of all women participating at T1, 413 (42.3%) were primiparae.

Follow-up was conducted by the study team approximately two months postpartum (T2). Women who provided an email address were sent a unique link to the questionnaire two months after their due-date using Qualtrics survey software. Women who did not complete the survey were followed up by text message, a reminder email and a phone call. Women who did not use email were mailed a paper questionnaire with a return envelope. These women were contacted by phone prior to the mailing of the questionnaire and two weeks after to ensure it was received and filled out. Return rates of the second survey were high for the whole sample (80%) and even higher for the primiparae (*n* = 342, 82.8%).

### Instruments

*Socio-demographics and obstetric history* were assessed at T1 and included socio-demographic questions such as age, educational level, income level, and obstetric history questions such as past pregnancy loss, fertility treatments, and pregnancy risk.

*Birth Beliefs* were assessed at both times using the Birth Beliefs Scale (BBS) [6] which includes 11 items in two subscales: Birth Belief Scale-Natural (BBS-Natural)–five items indicating that the birth process is normal and safe and should not be interfered with, for example: "A woman's body knows how to birth"; Birth Belief Scale-Medical (BBS-Medical)–six items indicating birth is a risky, dangerous process, and that women do not need to suffer the pain of birth, for example: "Birth requires rigorous medical attention". Women were asked to rate their agreement with each statement on a 1–5 Likert scale. Internal reliability for both subscales was sufficient at both time points (BBS-Medical–T1 α = 0.78, T2 α = 0.83; BBS-Natural–T1 α = 0.68, T2 α = 0.71). Scores were computed as subscale averages, with higher scores indicating a stronger belief.

*Actual Birth* was assessed at T2. Women were asked where and how they birthed, with the following options: Emergency CD; Planned CD; Instrumental VD; VD with epidural analgesia; VD without epidural analgesia in a regular delivery room; VD in a natural birth center; Home birth. A panel of three obstetricians and four midwives assessed these options for face validity and rated them on a medical to natural continuum. There ratings had an almost perfect agreement [12]. Therefore, the variable categories were ordinal (1 = most medical, emergency CD, to 7 = most natural, home birth).

*Birth Satisfaction* was assessed at T2 using the Childbirth Satisfaction Scale [19]. The scale includes 8 items assessing subjective general satisfaction with the birth experience such as "I am satisfied with the way I delivered" and "I wish my labor and delivery had gone differently than they did" (reverse scored). The scale was translated for the current study using forward-and-back translation by two experts fluent in both English and Hebrew. Women were asked to rate their agreement with the statements on a 1–5 Likert scale. The scale was unidimensional and internally reliable (α = 0.93). Scores were computed as scale averages, with higher scores indicating greater satisfaction.

### Statistical analyses

Analyses were performed using SPSS 24 [22] on the data set which is also provided as Supporting Information (S1 File). We used Pearson's correlations for univariate analyses and paired samples *t*-test for prenatal to postpartum changes. Hierarchical linear regression and the PROCESS macro [23] were used to predict changes in the birth beliefs: We predicted each T2 birth belief from its T1 value, Actual Birth, and Birth Satisfaction, and assessed the indirect effects of the birth experience variables as mediators of the association between the prenatal and postpartum beliefs.

## Results

Participants were on average 30.0±4.5 years old at recruitment (T1). They were mostly Jewish (98.5%) and married or cohabiting (95.3%). Additional socio-demographic characteristics can be found in Table 1.

**Table 1:** Socio-demographic characteristics of participants (*N* = 342).

Socio-demographics	*n* (%)	Obstetric history	*n* (%)
Income		Previous pregnancy loss	
Below average	51 (15.1)	No	272 (80.5)
Average	175 (51.8)	Yes	66 (19.5)
Above average	112 (33.1)	Fertility treatments	
Education		No	298 (88.2)
High school	35 (10.2)	Yes	40 (11.8)
Professional school	39 (11.4)	Pregnancy risk	
Undergraduate	190 (55.6)	Low risk	296 (86.8)
Graduate	78 (22.8)	High risk	45 (13.2)
Country of origin			
Israel	295 (86.3)		
Other	47 (13.7)		

Note: income levels were self-reported, women were asked if they had Below, Average or Above average income; Obstetric history was also self-reported (women were asked if they had previously lost a pregnancy, if they received fertility treatments to conceive the current pregnancy, and if the pregnancy was considered Low-risk or High-risk).

Descriptive statistics for the study variables are presented in Table 2. It is noticeable that in both timepoints, BBS-Natural had a higher mean and smaller standard deviation than the BBS-Medical indicating that women tended to view birth as a natural process, with more variance regarding birth as a medical process. The Actual Birth and Birth Satisfaction also had great variability. The most common Actual Birth was VD with epidural (*n* = 142, 41%), followed by instrumental birth (*n* = 64, 19%), emergency CD (*n* = 40, 12%), and VD without epidural in a regular delivery room (*n* = 38, 11%). Fewer women had an elective CD (*n* = 26, 8%), home birth (*n* = 19, 5%) or birth in a natural birth center (*n* = 13, 4%).

**Table 2:** Descriptive statistics of the main study variables.

Scale	Mean	Standard deviation	Actual range
Birth Belief Scale -Natural (T1)	4.09	0.59	2.20–5.00
Birth Belief Scale -Natural (T2)	3.94	0.64	1.60–5.00
Birth Belief Scale -Medical (T1)	3.30	0.84	1.17–5.00
Birth Belief Scale -Medical (T2)	3.39	0.83	1.17–5.00
Birth Satisfaction	3.80	1.03	1.00–5.00

Note: Possible range for all the scales was 1.00–5.00; T1- prenatal measurement; T2- two months postpartum.

Intercorrelation between study variables are presented in Table 3. The findings indicate that prenatal and postpartum BBS-Natural are positively correlated with Actual birth and with Birth Satisfaction. In other words, stronger beliefs about birth as a natural process were associated with a more natural objective delivery and with a more positive subjective birth experience. On the other hand, prenatal and postpartum BBS-Medical were negatively correlated with Actual Birth and with Birth Satisfaction. In other words, stronger beliefs that birth is a medical process were associated with a more medicalized objective birth and with a less satisfying subjective experience.

**Table 3:** Intercorrelations among the study variables.

	1	2	3	4	5
1. Birth Belief Scale -Natural (T1)	—				
2. Birth Belief Scale -Natural (T2)	0.63***	—			
3. Birth Belief Scale -Medical (T1)	-0.46***	-0.47***	—		
4. Birth Belief Scale -Medical (T2)	-0.52***	-0.49***	0.78***	—	
5. Actual Birth^a^	0.28***	0.65***	-0.36***	-0.45***	—
6. Birth Satisfaction	0.14*	0.33***	-0.16**	-0.24***	0.55***

*p < 0.05

**p < 0.01

***p < 0.001

^a^ Actual birth was coded from medical to natural (1 = emergency cesarean delivery, 7 = Home birth).

As can be seen, the associations between prenatal and comparable postpartum beliefs were strong (*r* = 0.63 and *r* = 0.78, respectively for the Natural and the Medical beliefs). Yet, when conducting a Students' *t*-test for paired variables, on average, women's postpartum BBS-Natural (*M* = 3.94±0.64) was weaker compared to the prenatal measurement (*M* = 4.10±0.58) (*t*(322) = 5.72, *p* < 0.001). Correspondingly, postpartum BBS-Medical (*M* = 3.39±0.83) was stronger compared to the prenatal measurement (*M* = 3.27±0.83) (*t*(323) = -4.01, *p* < 0.001). In other words, following birth, on average, the beliefs became more medical and less natural.

Hierarchical linear regression models were used to predict the effects of the birth experience on prenatal to postpartum changes in both the beliefs (Table 4). The dependent variable was the postpartum belief. In the first step of each regression model we entered the comparable prenatal Birth Belief and in the second step we added Actual Birth and Birth Satisfaction. By controlling for the prenatal BBS-Natural or BBS-Medical we were able to capture *changes* between the prenatal to postpartum period. By entering the two birth experience constructs simultaneously we could assess their unique contributions to the prenatal to postpartum changes in the beliefs. The results indicated that the BBS-Natural and BBS-Medical were influenced differently by Birth Satisfaction and Actual Birth. When predicting postpartum BBS-Natural, birth Satisfaction (but not Actual Birth) predicted an increase in postpartum BBS-Natural. In contrast, when predicting postpartum BBS-Medical, after controlling for the prenatal BBS-Medical, Actual Birth (but not Birth Satisfaction) predicted an increase in BBS-Medical.

**Table 4:** Linear regression models predicting prenatal to postpartum changes in basic birth beliefs (*n* = 322).

Dependent Independent	T2 Birth Belief Scale Natural		T2 Birth Belief Scale Medical	
	Step 1	Step 2	Step 1	Step 2
	*β*	*β*	*β*	*β*
T1 Birth Belief	0.63***	0.58***	0.72***	0.72***
Actual Birth		0.07		-0.18***
Birth Satisfaction		0.22***		-0.02
R^2^	0.39	0.46	0.62	0.65
ΔR^2^		0.07		0.03
*F*	208.98***	90.53***	516.41***	197.28***
Δ*F*		19.35***		15.10***

Note: T1- prenatal measurement; T2- two months postpartum.

***p < 0.001

**Fig 1:**
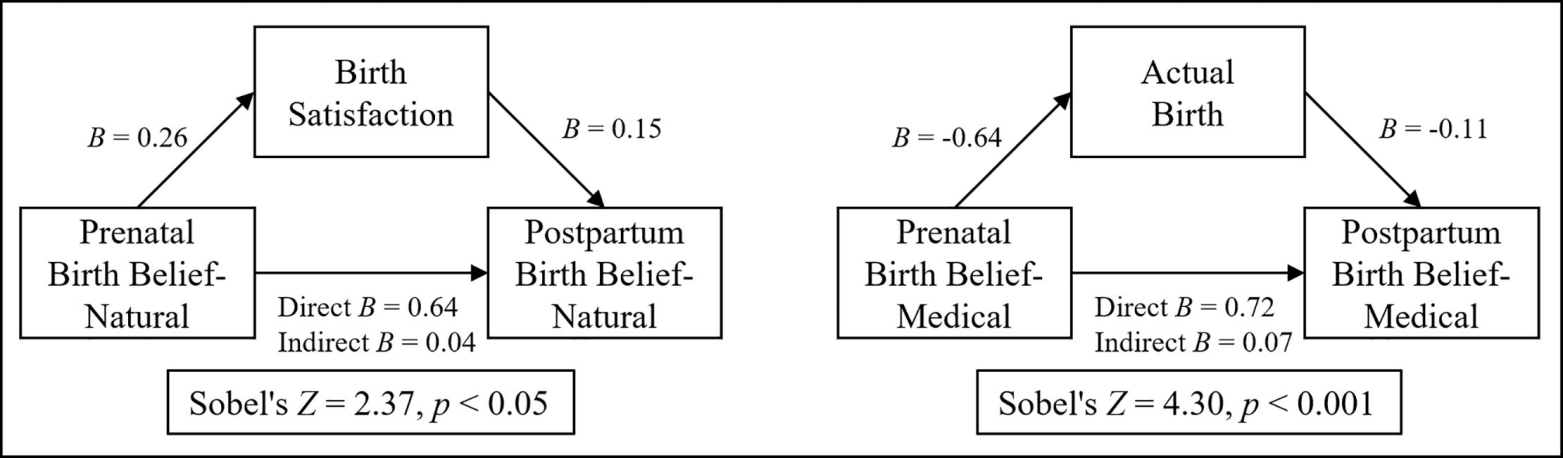
Mediation effects of Actual Birth and Birth Satisfaction.

Since there were correlations between the prenatal BBS-Natural and Birth Satisfaction and BBS-Medical and Actual Birth, there was reason to examine whether the birth experiences mediated the association between the prenatal and postpartum beliefs. Mediation tests conducted with the PROCESS macro indicated significant partial mediation (Fig 1): significant paths from BBS-Natural through having a more satisfying birth to a higher postpartum BBS-Natural and similarly, from BBS-Medical to having a more medicalized Actual Birth, which further increased the BBS-Medical.

## Discussion

Women approaching their first birth mostly agreed that birth is a natural process yet were more varied regarding the belief that it is a medical process. These findings are in line with those from qualitative studies showing that although women in Western, medicalized birth cultures [24] are often bombarded with messages regarding the riskiness of birth [25], views of the naturalness of birth are still prevalent [26]. Following their lived, often medicalized, birth experience, women's belief that birth is a natural process decreased and their belief that it is medical process increased. In both models, when simultaneously examining the objective and the subjective birth experience, only one experience and not the other was predictive of changes in the beliefs. These findings highlight the importance of recognizing the complexity of the birth experience [27,16], paying attention to both the objective and subjective reality [3], and differentiating between the two beliefs [6,11].

The mediation model indicated that women who tended to view birth as medical and dangerous also tended to have more medicalized births (along a wide range of birth choices), which in turn perpetuated and even strengthened their medicalized beliefs. Modern birth culture emphasizes the inherent faultiness of the female body [28] and in a sense, these women's prophecy regarding the riskiness of birth came true. Thus, reaffirming their belief that it should be managed by medical staff with the use of the latest technology, because of the likelihood of something going wrong.

Beliefs about birth as a natural process were associated with birth satisfaction in a reciprocal manner. Women who prenatally had stronger beliefs that birth is a natural process, were more satisfied with their births, which led to even stronger beliefs that it is a natural process. It is possible that the natural beliefs affected their expectations and experience of birth [5,13,14]. Women with a more natural belief may anticipate a more positive experience as they understand that childbirth is often uncontrollable [29]. They also view pain as intrinsic to the birth process [14] and it is less perceived as unpleasant [30]. Among these women, a satisfying (more natural) experience was empowering–reinforcing their belief in themselves and in their body [31,32].

The knowledge gained from this study could help practitioners who provide perinatal care to women, better relate to women's birth expectations and experiences. As obstetrics shifts to more patient-centered care, there is increasing interest in women's perceptions and needs [33]. Birth attendants' awareness of the potential effects that the birth beliefs can have on the birth experience and the way birth experiences affect the beliefs, could improve their understanding of their patient's inner world. Prenatally, practitioners could communicate with women about their birth beliefs, help them achieve their expected birth, and, by emphasizing the naturalness of giving birth, promote more satisfactory birth. Postpartum, practitioners can empower women whose birth experience was different than planned: When birth was unsatisfactory, they could help maintain the belief that birth is natural and when it was highly medicalized, they could try to prevent an increase in the belief that birth is medical and risky. Such practice, for example, could decrease the risk of a medicalized second birth, such as repeat CD, and encourage trial of labor, when medically possible [6].

One of the strengths of our study was that it was prospective, measuring beliefs both before and after women's first birth. Thus, we could estimate change in our outcome measures related to the birth experience, though causality could not be conclusively determined. Additional strengths included the sample size, focusing on first-time mothers, and the heterogeneity of birth modes. However, despite the rigor of the sampling method and the large sample size, it was not representative of the general Israeli population and generalization should be made with caution.

Our conclusion is in line with the recent World Health Organization guidelines regarding encouraging intrapartum care practice which contributes to a positive birth experience [34]: Supporting safe physiological birth, avoiding unnecessarily medicalized births [35,36] and improving satisfaction with birth should be a main goal for maternity care providers. Additionally, psychosocial professionals should aim to decrease women's fears, and strengthen their self-efficacy. Doing these would empower women, increasing their beliefs in themselves, their bodies, and the natural course of birth.

## Supporting information

S1 File Minimal data file- changes in birth beliefs following first birth. (XLSX)
